# Lack of progression of beta dynamics after long‐term subthalamic neurostimulation

**DOI:** 10.1002/acn3.51463

**Published:** 2021-10-11

**Authors:** Ross W. Anderson, Kevin B. Wilkins, Jordan E. Parker, Matthew N. Petrucci, Yasmine Kehnemouyi, Raumin S. Neuville, Declan Cassini, Megan H. Trager, Mandy M. Koop, Anca Velisar, Zack Blumenfeld, Emma J. Quinn, Jaimie Henderson, Helen M. Bronte‐Stewart

**Affiliations:** ^1^ Department of Neurology and Neurological Sciences Stanford University School of Medicine Stanford California USA; ^2^ Department of Neurosurgery, Kaiser Permanente Redwood City California USA; ^3^ Department of Psychology The University of California Los Angeles California USA; ^4^ Department of Bioengineering Stanford Schools of Engineering & Medicine Stanford California USA; ^5^ The University of California School of Medicine Irvine California USA; ^6^ Columbia University College of Physicians and Surgeons New York City New York USA; ^7^ Cleveland Clinic Cleveland Ohio USA; ^8^ The Smith‐Kettlewell Eye Research Institute San Francisco California USA; ^9^ California Institute of Technology Pasadena California USA; ^10^ Credit Karma San Francisco California USA; ^11^ Department of Neurosurgery Stanford University School of Medicine Stanford California USA

## Abstract

**Objective:**

To investigate the progression of neural and motor features of Parkinson's disease in a longitudinal study, after washout of medication and bilateral subthalamic nucleus deep brain stimulation (STN DBS).

**Methods:**

Participants with clinically established Parkinson's disease underwent bilateral implantation of DBS leads (18 participants, 13 male) within the STN using standard functional frameless stereotactic technique and multi‐pass microelectrode recording. Both DBS leads were connected to an implanted investigative sensing neurostimulator (Activa™ PC + S, Medtronic, PLC). Resting state STN local field potentials (LFPs) were recorded and motor disability, (the Movement Disorder Society‐Unified Parkinson's Disease Rating Scale – motor subscale, MDS‐UPDRS III) was assessed off therapy at initial programming, and after 6 months, 1 year, and yearly out to 5 years of treatment. The primary endpoint was measured at 3 years. At each visit, medication had been held for over 12/24 h and DBS was turned off for at least 60 min, by which time LFP spectra reached a steady state.

**Results:**

After 3 years of chronic DBS, there were no increases in STN beta band dynamics (*p* = 0.98) but there were increases in alpha band dynamics (*p* = 0.0027, 25 STNs). Similar results were observed in a smaller cohort out to 5 years. There was no increase in the MDS‐UPDRS III score.

**Interpretation:**

These findings provide evidence that the beta oscillopathy does not substantially progress following combined STN DBS plus medication in moderate to advanced Parkinson's disease.

## Introduction

Parkinson's disease is a progressive neurological disease, whose severity worsens even on optimal doses of medication.^1^, ^2^, ^3^ Recordings of local field potentials (LFPs) in the subthalamic nucleus (STN) have revealed exaggerated neuronal oscillations and synchrony in the beta frequency band (13–30 Hz), known as the beta oscillopathy, which is a marker of the pathophysiology of Parkinson's disease.^4^, ^5^, ^6^, ^7^ Beta power has been linked to motor impairment and longer duration of fluctuations of beta power (bursts) are associated with increased motor and gait disablity.^7^, ^8^, ^9^, ^10^, ^11^, ^12^, ^13^, ^14^ Increasing beta power over time is linked to progressive Parkinsonian pathophysiology in neural recordings from both non‐human primate and rodent models of progressive Parkinsonism.^15^, ^16^, ^17^, ^18^, ^19^ Although a causal link between beta power and PD impairment in humans is still lacking, resting state beta power is greater in the more affected compared to the lesser affected STN,^6^ and beta power has been found to increase over time in the untreated STN in two individuals with Parkinson's disease, further suggesting a relationship to disease progression.^20^

Beta power was attenuated and burst durations were shortened during short periods of high frequency STN DBS in a dose‐dependent manner while improving motor disability^14^, ^21^, ^22^ and for a period after STN DBS was turned off,^23^, ^24^, ^25^ particularly when neurostimulation targeted the sensorimotor portion of the STN.^7^, ^26^ Meanwhile, investigation of the long‐term therapeutic efficacy of STN DBS on the beta oscillopathy^20^ has been limited to the first 12 months after the start of STN DBS.^20^, ^27^, ^28^ Initial results from these studies suggest that the beta oscillopathy does not substantially progress during the initial 6–12 months of DBS.

Several studies have documented a lack of significant progression of overall off therapy MDS‐Unified Parkinson's Disease Rating Scale part III (MDS‐UPDRS III) scores after long‐term STN DBS, in contrast to the progression of motor signs on and off long‐term medical therapy.^20^, ^29^, ^30^, ^31^, ^32^, ^33^, ^34^ When examining specific symptoms, gait, balance, and speech may continue to worsen over time,^35^, ^36^, ^37^ whereas tremor has been shown to be arrested in early stage PD after 2 years of combined STN DBS plus medication compared to treatment with medication alone.^29^, ^33^ However, it is unknown if the long‐term stabilization of overall motor disability following chronic STN DBS is accompanied by a stabilization of the STN beta oscillopathy.

In this study, we tracked the dynamics of the off therapy beta oscillopathy after 3–5 years STN DBS, using the largest cohort of longitudinal data to date. Resting state STN LFPs and the MDS‐UPDRS III were measured off therapy (i.e., after withdrawal of medication and STN DBS) every 6 months out to 5 years of chronic STN DBS. We hypothesized that there would be no significant progression of the beta oscillopathy, as measured by both beta power and burst duration, nor overall motor disability following withdrawal of chronic STN DBS. In contrast, the beta oscillopathy and motor signs would continue to progress in a control STN, in which a DBS lead was implanted but not turned on, and then stabilize after activation of DBS.

## Materials and Methods

### Human subjects

Eighteen participants (13 male) with clinically established Parkinson's disease (Table 1) underwent bilateral implantation of DBS leads (model 3389, Medtronic, PLC, Minneapolis, MN, USA) in the sensorimotor region of the subthalamic nucleus (STN) using a standard functional frameless stereotactic technique and multi‐pass microelectrode recording. Dorsal and ventral borders of each STN were determined using microelectrode recording, and the base of electrode zero was placed at the ventral border of the STN. The two leads were connected to the implanted investigative neurostimulator (Activa™ PC + S, Medtronic, PLC). The preoperative selection criteria and surgical technique have been previously described.^38^ All experimental testing was done in the off‐medication state, which entailed stopping long‐acting dopamine agonists at least 48 h, dopamine agonists and controlled release carbidopa/levodopa at least 24 h, and short acting medication at least 12 h before testing. To be eligible for analysis in this long‐term washout study, individuals implanted with the Activa™ PC + S sensing neurostimulator had to have at least a 12‐month washout data point.^20^ All participants gave written consent to participate in the study, which was approved by the Food and Drug Administration (FDA) with an investigational device exemption (IDE) and by the Stanford University Institutional Review Board (IRB).

**Table 1:** Participant demographics.

Participant	Age	Sex	Disease duration at IP	Pre‐Op UPDRS III (off meds)	Pre‐Op UPDRS III (on meds)	IP MDS‐UPDRS III (off therapy)	1‐year MDS‐UPDRS III (ON DBS, off meds)	IP LEDD (mg)	1‐year LEDD (mg)	3‐year LEDD (mg)
1	52.8	M	4.9	24	8	20	5	1314	100	0
2	62.5	M	4.0	35	26	36	11	400	550	75
3	65.7	M	7.2	29	16	38	12	500	500	125
4	58.1	M	7.3	39	23	43	19	1923	650	650
5	42.2	M	7.5	58	12	51	9	1200	550	200
6	53.5	M	7.6	52	29	61	17	2405	850	550
7^a^	72.2	M	9.9	47	23	45	29	750	400	275
8^b^	58.6	F	11.0	33	6	42	18	1000	225	175
9	34.3	M	8.1	59	22	70	35	950	250	0
10^c^	57.3	M	6.1	62	23	56	19	1075	750	–
11	67.6	F	7.1	44	25	12	2	825	500	200
12	50.6	F	11.2	50	26	51	14	920	660	0
13^c^	61.9	M	17.3	42	24	44	8	1200	150	–
14	52.0	F	12.5	34	6	22	6	650	1980	707
15	66.1	M	8.6	51	10	33	16	875	100	350
16	56.8	F	13.6	38	18	35	8	1125	550	350
17^c^	55.0	M	12.3	18	4	21	2	917	100	–
18^c^	50.1	M	11.2	38	31	48	17	375	100	–
Average ± SD	56.5 ± 8.9		9.3 ± 3.4	41.8 ± 11.8	18.4 ± 8.4	40.4 ± 15.2	13.7 ± 8.5	1022.4 ± 486.7	498.1 ± 430.9	261.1 ± 227.1

LEDD, levodopa equivalent daily dose; IP, initial programming.

^a^ Indicates participant had a broken right hand and could not perform complete UPRDS at 3‐year follow‐up.

^b^ Participant did not perform the gait task of the UPRDS at 3‐year follow‐up.

^c^ Indicates that the participant did not perform the 3‐year follow‐up.

### Experimental protocol

Recordings were collected before the initial activation of the DBS system at initial programming (IP), which was 1 month after implantation of the DBS leads, and off all therapy after 6 months, and then 1, 2, 3, 4, and 5 years after the initial programing (IP) visit. At the follow‐up visits, stimulation was turned off and seated resting state local field potential (LFP) recordings were collected 60–75 min later. This timing was chosen since we have previously demonstrated that the effect of DBS on the LFP is washed out well before this time window.^20^ Limb or head movement was monitored using angular velocity sensors on the hands and feet (Motus Bioengineering, Inc., Benicia, CA), a triaxial accelerometer on the forehead (ADXL335, Analog Devices, Norewood, MA), and with synchronized video recordings (30 FPS) from a USB web camera (C930e, Logitech, Lausanne, Switzerland). The MDS‐UPDRS III – motor subscale was performed by a certified rater preoperatively (on and off medication), off medication at IP, and then off all therapy at all follow‐up visits after DBS had been turned off for at least 60 min.

### Data acquisition and analysis

#### LFP data acquisition

STN LFPs were recorded from electrode contact pair 0–2 or 1–3 on the DBS leads. LFPs were high‐pass filtered at 0.5 Hz, low‐pass filtered at 100 Hz within the device, and sampled at 422 Hz (10‐bit resolution). Uncompressed neural data were recorded on to the Activa™ PC + S system and then extracted via telemetry using the Activa™ PC + S tablet programmer.

#### Data analysis

Estimated power spectral density was calculated using Welch's method with a 1‐sec sliding Hanning window with 50% overlap on 30 sec of resting state data. Power was calculated across the whole alpha (8–12) and beta band (13–30 Hz) and normalized by dividing these values by the mean power across the 45–65 Hz band, enabling comparison among STNs.^39^

#### Oscillatory dynamics

Fluctuations in beta band power, characterized as bursts, were measured in reference to a physiological baseline (i.e., the 1/*f* spectrum characteristic of broadband neural activity) that captures a broad distribution of fluctuations.^14^ To calculate beta bursts, the raw LFP was filtered with a zero‐phase 8th order Butterworth bandpass filter with either a 4 Hz band spanning the alpha band, a 17 Hz bandwidth spanning the whole beta band (13–30 Hz), or 6 Hz bandwidth centered around the peak beta frequency.^14^ The bandpass filtered signal was squared and an amplitude envelope of the maximum power was created by linearly connecting consecutive peaks of the squared LFP signal. The baseline for thresholding was established by filtering the LFP between 45 and 65 Hz in sequential, 50% overlapping bands of the bandwidth used in the analysis, then squaring, and creating an envelope of that signal. A trough detection algorithm identified the local minima of the envelope, which represented the local smallest peak amplitudes of the filtered squared signal. The threshold to be used as the baseline to identify alpha or beta band bursts was established as 4 times the median power of the troughs of the LFP, as described previously.^14^ Burst durations were calculated as the interval between successive crossings of the envelope of the band of interest over the baseline. Burst peak power was calculated as the maximum excursion of the power envelope during a burst. In one STN, the alpha band envelope power was elevated in the 3‐year follow‐up visit and failed to drop below the threshold for defining bursts and consequently no burst was detected. This STN was excluded from the analysis.

#### Localization of DBS leads

Preoperative T1 and T2 MRI scans and postoperative CT scans were acquired as part of the standard clinical protocol.^38^ Location of DBS leads was determined by the Lead‐DBS toolbox.^40^ Postoperative CT scans and preoperative T1 and T2 scans were co‐registered, which were then normalized into MNI space using SPM12 (Statistical Parametric Mapping 12; Wellcome Trust Centre for Neuroimaging, UCL, London, UK) and Advanced Normalization Tools.^41^ DBS electrode localizations were then corrected for brain‐shift in the postoperative CT scan.^40^ DBS electrodes were then localized in template space using the PaCER algorithm^42^ and projected onto the DISTAL Atlas to visualize overlap with the STN.^43^

#### Calculation of volume of tissue activated

Volumes of tissue activated (VTA) were modeled using the approach described by Horn and colleagues.^40^ Electric fields were estimated using a finite element model with a four‐compartment tetrahedral mesh segmenting gray matter, white matter, electrode contacts, and insulating materials. The percent volume of overlap was then computed for both the whole STN and the sensorimotor portion of the STN based on the DISTAL Atlas.^43^ Additionally, the overlapping VTA that was common across all STNs (left and right separately) was calculated.

### Data acquisition and analysis

Statistics were computed using MATLAB (version 9.9, The MathWorks Inc. Natick, MA). Paired *t*‐tests were used to assess changes in power, burst duration, and burst peak power, as well as in the MDS‐UPDRS III from IP to the 3‐year timepoint. Spearman correlations were used to assess any correlation between percent change in MDS‐UPDRS III and the percent change of each of the neural metrics with each STN treated independently. Significance was set at *p* < 0.05.

## Results

The primary outcomes were the change in beta band power and the MDS‐UPDRS III from IP to 3 years of continuous high frequency STN DBS. Eighteen individuals (33 STNs) completed the 1‐year evaluation, 14 individuals (25 STNs) completed the 2‐year evaluation, 14 individuals (25 STNs) completed the 3‐year evaluation, 9 individuals (16 STNs) completed the 4‐year evaluation, and four individuals (6 STNs) completed the 5‐year evaluation. Decrement in enrollment at later visits was primarily from lack of re‐implantation of the experimental neurostimulator (PC + S) or from late enrollment in the study. The mean age of the participants was 56.5 ± 8.9 years, and mean disease duration at IP was 9.3 ± 3.4 years (Table 1).

### DBS lead location accurately targets the sensorimotor STN

**Figure 1:**
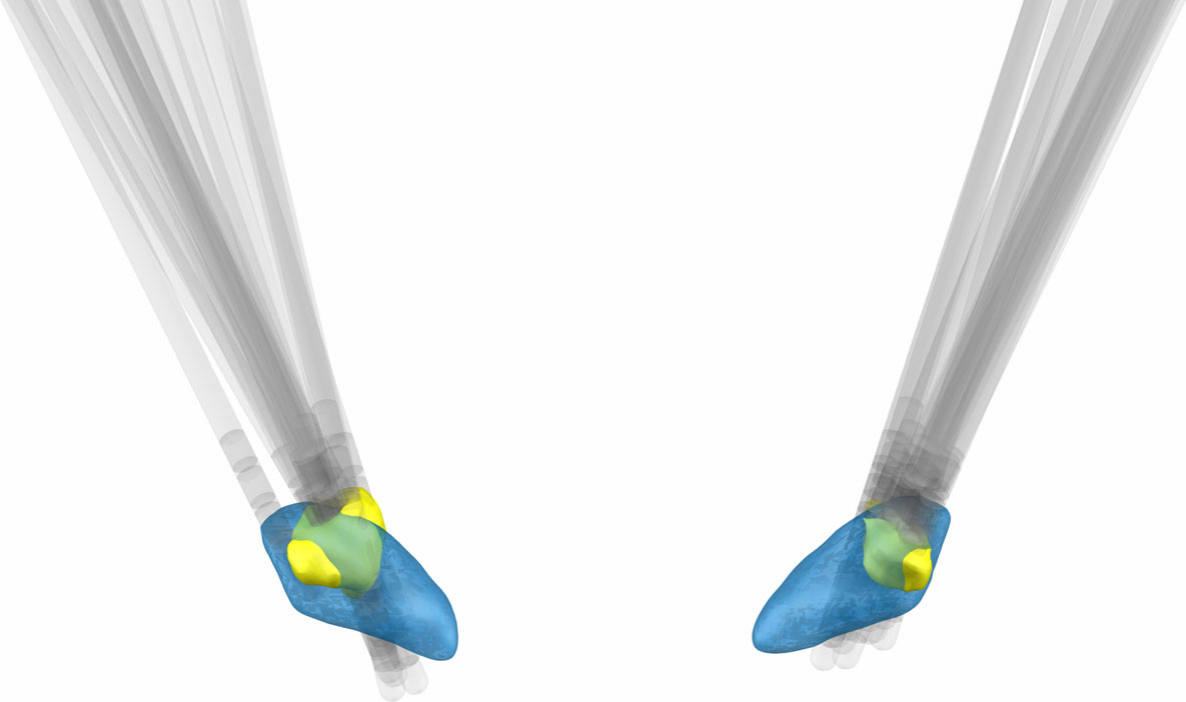
Lead location and shared volumes of tissue modulated (VTAs). (A) Lead location for all 18 participants within the subthalamic nucleus (blue). (B) Depiction of the VTAs (yellow) at the 3‐year timepoint that are shared across all participants.

Figure 1 demonstrates that the DBS lead locations for all subjects were well placed in the STN, and that the shared VTA of the participants included in the 3‐year analysis overlapped the dorsolateral (sensorimotor) region of the STN (average overlap ± standard deviation: 61.6% ± 17.5%, range: 26.8%–99.9%).

### STN DBS improved motor disability and resulted in reduction of medication

Table 1 demonstrates that there were significant reductions in the MDS‐UPDRS III on compared to off medication preoperatively, and ON DBS at the 1‐year timepoint compared to off medication preoperatively: preoperative off and on medication MDS‐UPDRS III scores were 41.8 ± 11.8 and 18.4 ± 8.4, (*t*(17) = 8.87, *p* = 8.67–8) and 13.7 ± 8.5, off medication, ON DBS after 1 year of DBS (*t*(17) = 11.51, *p* = 1.89–9). For those with data out to 3 years, the levodopa equivalent daily dose (LEDD) decreased 75.4% from 1059.8 ± 539.3 mg at IP to 261.2 ± 235.7 mg at 3 years (*p* = 3.28e‐5), with all but one participant showing reduced LEDD. Three/fourteen (21%) participants had not been taking any dopaminergic medication for at least 9 months at the 3‐year timepoint.

### Off therapy beta band power was unchanged while alpha band power increased after 3 years of DBS

**Figure 2:**
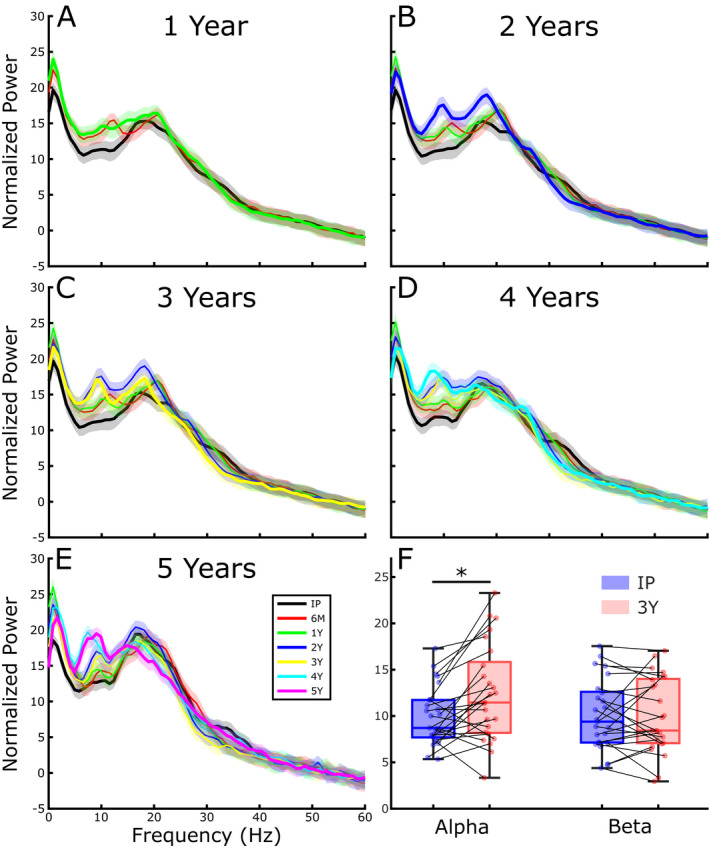
Off therapy grand average STN LFP power spectral density analysis before (IP) and at six different timepoints after activation of DBS: IP, 6 months, 1 year (A, 33 STNs), including 2 years (B, 25 STNs), including 3 years (C, 25 STNs), including 4 years (D, 16 STNs), and including 5 years (E, 6 STNs). (F) Quantification of alpha (8–12 Hz) and beta (13–30 Hz) spectral bands at IP and after 3 years of DBS (*p = 0.0027).

Figure 2 displays the grand average resting state power spectral density, measured off therapy, at up to seven timepoints out to 5 years after the IP visit. Visual observation suggested that there was no increase in beta band power over time after up to 5 years of STN DBS, whereas alpha band power increased, Figure 2A–E. This was quantified between the IP and the 3‐year timepoint in 25 STNs, where there was no change in beta power (*t*(24) = 0.027, *p* = 0.98) and a significant increase in alpha power (*t*(24) = 3.35, *p* = 0.0027), Figure 2F. The lack of change in beta band power and increase in alpha band power was observed in both akinetic rigid phenotype patients and in tremor dominant patients.

**Figure 3:**
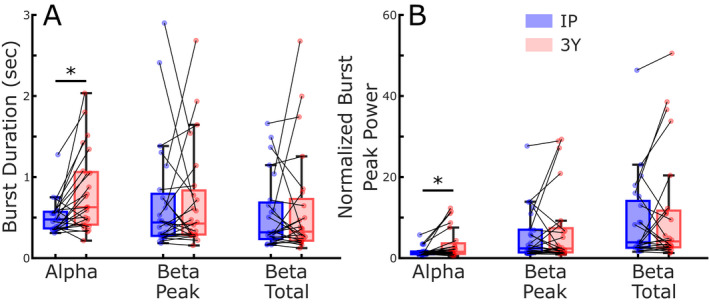
Off therapy burst duration (A) and burst peak power (B) at initial programing and after 3 years of DBS. Burst durations and peak power were calculated for the alpha band (8–12 Hz), for the participant specific 6‐Hz beta band centered at the peak beta power (beta peak) and for the total beta band (13–30 Hz, beta total), *p = 0.009.

Oscillatory dynamics were quantified by burst duration and normalized burst peak power. Alpha band burst durations were significantly longer off therapy after 3 years of STN DBS, compared to IP, Figure 3A (*t*(23) = 3.53, *p* = 0.0018). However, there was no difference in mean burst duration in the beta band, either for a 6 Hz narrowband centered around the beta peak (*t*(23) = 0.01, *p* = 0.99), or for the whole 13–30 Hz beta band (*t*(23) = 0.45, *p* = 0.65). Similarly, alpha peak power was significantly greater at the 3‐year timepoint, Figure 3B (*t*(23) = 2.85, *p* = 0.009). Meanwhile, there was no change in beta peak power for either the narrowband (*t*(23) = 1.13, *p* = 0.27) or whole beta band (*t*(23) = 1.28, *p* = 0.21).

### Off therapy overall motor disability did not progress after 3 years of STN DBS

Fourteen individuals were assessed at both IP and the 3‐year timepoint with 12 individuals able to complete the full MDS‐UPDRS III at follow‐up (one had a broken right hand and one could not perform the gait task) to assess whether motor signs changed over time, off all therapy. Off therapy motor disability, assessed by the total MDS‐UPDRS III score, did not worsen after washout of 3 years of continuous STN DBS plus medication compared to the initial programming (IP) off therapy scores (IP: 39.3 ± 17.0; 3‐years: 39.8 ± 21.4) after washout of medication and before activation of DBS (*t*(11) = 0.11, *p* = 0.92).

There were no significant correlations between percent change in UPDRS scores and the percent change in beta power (*ρ* = −0.09, *p* = 0.69), beta burst duration (*ρ* = 0.23, *p* = 0.32), or beta peak power (*ρ* = 0.05, *p* = 0.83). Similarly, there was no significant correlation with percent change in alpha power (*ρ* = 0.01, *p* = 0.98), alpha burst duration (*ρ* = 0.19, *p* = 0.40), or alpha peak power (*ρ* = 0.20, *p* = 0.38).

### Progression of beta in the untreated side

**Figure 4:**
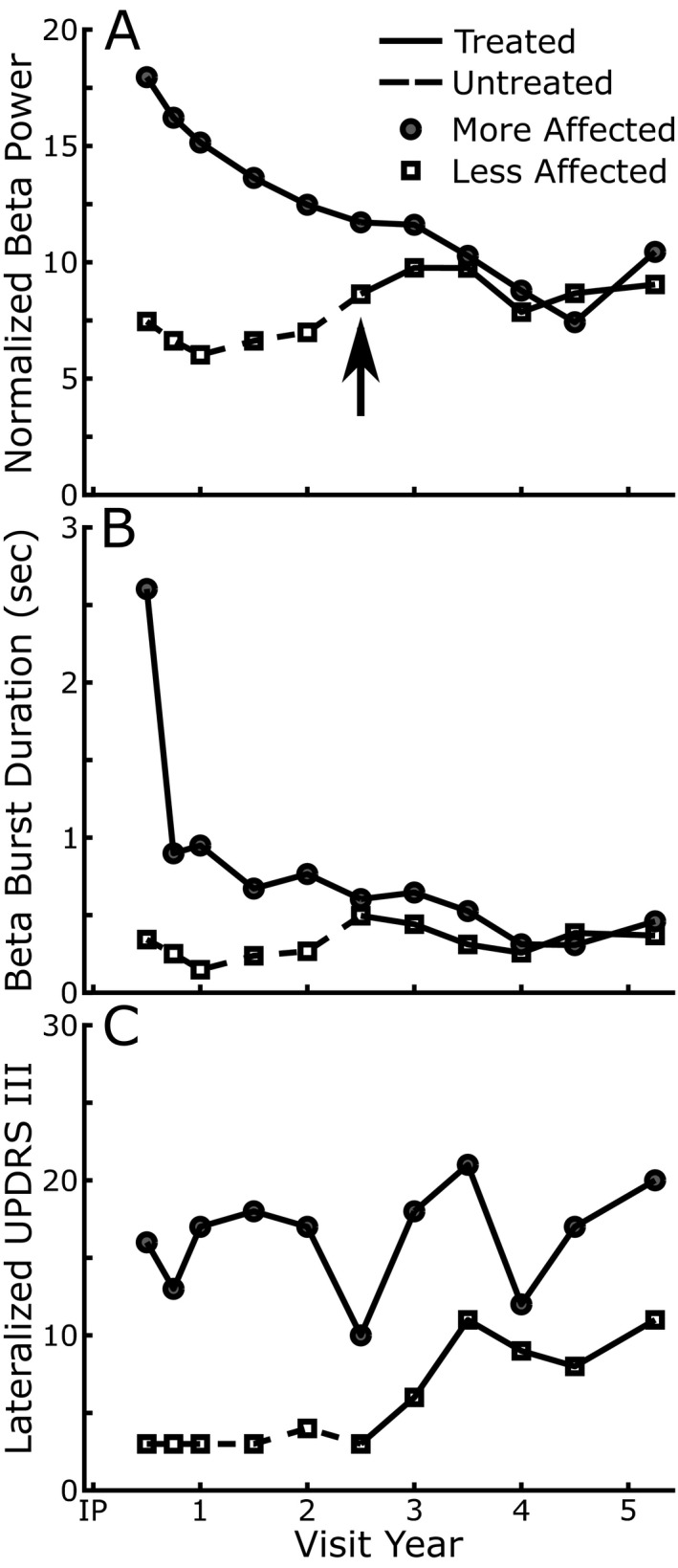
STN beta band power, burst duration, and lateralized MDS‐UPDRS III scores over time in a single patient. Dashed lines represent the period when the less affected STN was not receiving DBS therapy. (A) Normalized STN beta band power, (B) STN beta band burst durations, and (C) modified lateralized off therapy MDS‐UPDRS III scores (tremor, bradykinesia, and rigidity) over time for the more (closed circles) and less affected (open squares) STNs.

One participant chose to have bilateral DBS lead implantation, but initially only activated unilateral DBS for the more affected hemibody. The less affected side was turned on 30 months later based on a clinical decision due to the progression of symptoms. Figure 4 details the off‐therapy measurements of beta band power (Figure 4A), beta band mean burst duration (Figure 4B), and the MDS‐UPDRS III lateralized scores of tremor, rigidity, and bradykinesia (Figure 4C) of the more and less affected hemibodies. There was progressive attenuation of beta band power and shortening of mean beta burst durations in the more affected (treated) STN, after washout of therapy out to 5 years. In the less affected STN, beta band power and mean burst duration began to increase after 2 years of no DBS therapy. After STN DBS was turned on at 30 months, beta burst durations stabilized, Figure 4B. Meanwhile, the less affected hemibody lateralized MDS‐UPDRS III scores continued to increase at the 36‐ and 42‐month timepoint, but then stabilized, Figure 4C.

## Discussion

This longitudinal study, recording both STN neural activity and clinical motor disability, demonstrated that there was no increase (i.e., worsening) in STN beta band power, mean beta band burst duration, peak beta burst power, or the MDS‐UPDRS III motor score after a washout of both STN DBS and medication after 3 years. In contrast to the stabilization of beta band dynamics off therapy over time, there was a significant increase in alpha band power, mean alpha band burst duration, and peak alpha burst power. The stabilization of beta and increase in alpha band dynamics were also evident after 4 and 5 years of DBS in a smaller cohort. In a control STN the beta oscillopathy increased with no DBS therapy and then stabilized after activation of STN DBS.

### Lack of progression of beta oscillopathy following chronic STN DBS

In animal models of Parkinsonism, it has been demonstrated that increasing beta band power and longer beta bursts are markers of progressive Parkinsonian pathophysiology.^15^, ^16^, ^17^, ^18^, ^19^ There have been repeated demonstrations of the short‐term effect of therapy (both DBS and medication) on the beta oscillopathy.^7^, ^11^, ^44^ The only longitudinal studies to date tracking beta power have been limited to the first year post‐DBS and have each shown either a reduction or stability of beta over that time. Our results build on this by examining LFP data out to 3 years, along with observational support out to 5 years in a more limited cohort and finding no progression of the beta oscillopathy. This result was consistent for both akinetic rigid and tremor‐dominant subtypes. Although the lack of change in beta oscillopathy is not proof of stabilization, it does provide good evidence that beta is not significantly progressing for those receiving chronic STN DBS. We do not believe that the lack of significant progression of beta was due to the variable nature of LFP data, as alpha dynamics did show a consistent change over time, and the lack of beta change was consistent at each timepoint over 5 years. Additionally, in one control STN that was not initially turned on, we did see the beta oscillopathy start to progress after 2 years with medication as the sole treatment, which then stabilized following activation of DBS. However, as an N of 1, that result is only anecdotal.

In contrast to the lack of change in beta band dynamics, we observed an increase in alpha band power and prolongation of alpha band burst durations after washout of 3 years of STN DBS. We do not believe that this was due to emergence of tremor over time as these findings appeared similar in both tremor‐dominant and akinetic‐rigid subtypes. Unlike beta, the role of alpha within the STN is not well characterized. Topographic analysis of oscillatory activity within the STN has demonstrated that alpha peaks may reside more ventromedially within the STN compared to the dorsolateral location of beta oscillations, which may correspond with projections toward premotor and prefrontal areas.^45^ Additionally, cortical alpha oscillations have been shown to play a significant role in attentional‐processing.^46^ It is possible, therefore, that the observed changes in alpha may represent cognitive or attention‐related changes over the course of the disease. These are typically less well addressed by STN DBS compared to traditional motor symptoms.

### Chronic STN DBS slowed expected overall motor progression off therapy in treated Parkinson's disease

The progression of motor signs in the drug naïve state has been hard to measure in the era of levodopa therapy due to the long duration effect of dopaminergic medication, which has been reported to persist in advanced Parkinson's disease.^2^, ^47^, ^48^ However, the drug naïve state is not the expected motor state of Parkinson's disease, except in very early stages of disease, as demonstrated in the Parkinson's Progression Markers Initiative (PPMI) cohort. Of the 423 people with Parkinson's recruited in the drug naïve state (disease duration: 0.4–35.8 months) almost 50% had started levodopa therapy after 2 years, which increased to 83% at 5 years.^2^ Therefore, in order to assess the expected motor progression, the only comparison that can be made between the cohort in this paper and treated people with Parkinson's disease is in the off‐medication state. After withdrawal of medication, the MDS‐UPDRS III score increased by an average of 8.8 points in the PPMI cohort.^3^ Over several cohorts there appears to be an annual ˜2.4 point increase in the MDS‐UPDRS III score after withdrawal of medication^2^, ^47^ and even on optimal doses of medication, motor severity worsens over time.^1^, ^2^, ^3^, ^47^

In contrast, the off medication/OFF DBS MDS‐UPDRS III score in this study did not change after 3 years (IP: 39.3 ± 17.0, 3 years: 39.8 ± 21.4). There was a significant (75.4%) reduction in medication (LEDD) and three/fourteen participants had stopped medication for several months prior to their 3‐year evaluation. This argues against a contribution of the long duration effect of dopaminergic medication as the overall lower dose of medication and fewer participants on medication would predict an even greater increase in the MDS‐UPDRS III after 3 years. Other factors arguing for an expected progression of motor signs greater than that observed include a longer disease duration (9.3 + 3.4 years) at baseline than the PPMI cohort and that medication was withdrawn for a longer time. The lack of progression of off therapy overall MDS‐UPDRS III scores after chronic long‐term STN DBS has also been documented in previous studies,^20^, ^30^, ^31^, ^32^, ^34^ although it is important to note that certain symptoms such as axial signs may continue to worsen.^35^, ^36^, ^37^

### Implications for closed‐loop DBS

The stability of beta power and burst duration out to 5 years post DBS implantation is a critical observation for the potential feasibility of using beta as a control variable for closed‐loop DBS. Up until this point, it has not been known whether beta could reliably be used as a long‐term biomarker due to a lack of in‐human subject data in chronically implanted patients. The fact that beta remained stable out to 5 years in this cohort should assuage concerns over potential loss of signal quality over time.

### Limitations

MDS‐UPDRS III and neural recordings were made 60–75 min after DBS was turned off and at least 12 h after withdrawal of short acting dopaminergic medication and 24–48 h after long‐acting dopaminergic medication. Although tremor and rigidity have been shown to return to baseline within 60 min of turning off DBS, the return of axial, and bradykinetic symptoms have been shown to take 2–4 h.^49^ Similarly, the action of medications may take weeks to fully washout.^50^ However, the participants were on much less medication at the 3‐year timepoint which would bias the results toward less of a long duration medication effect at 3 years compared to that at IP and would therefore lead to a greater expected increase in MDS‐UPDRS III score. Meanwhile, we can be confident that the recorded washout LFPs are stable as we have previously demonstrated that there was no difference in beta power and the power spectral density spectrum between the 14‐sec and the 60‐min timepoint after turning off STN DBS.^20^

The lack of a control group means that we cannot rule out that the stabilization of the beta oscillopathy does not represent a normal plateau in Parkinson's disease. However, the observed worsening of the beta oscillopathy in the case example of the untreated STN, alongside observations of worsening in non‐human primate and rodent models of progressive Parkinsonism,^16^, ^18^ provide evidence that the observed stabilization in the STN DBS cohort may be connected to treatment. However, no correlation was seen between change in UPDRS and change in beta oscillopathy. More objective measurements of behavior in the future may allow for higher fidelity tracking of different symptom progression over time and allow us to better characterize any link to the beta oscillopathy or other neural features.

The results of this study demonstrate that the underlying, off therapy beta oscillopathy and overall motor disability of people with moderate to advanced Parkinson's disease did not substantially increase following 3–5 years of STN DBS, whereas alpha band power increased. These results are interesting in relation to evidence that the STN beta oscillopathy is a marker of progressive Parkinsonian pathophysiology.

## Author Contributions

RWA, KBW, MHT, MMK, ZB, EJQ, and HBS contributed to the conception and design of the study; RWA, KBW, JEP, MNP, YK, RSN, DC, MHT, MMK, AV, ZB, EJQ, JH, and HBS contributed to the acquisition and analysis of data; RWA, KBW, MNP, and HBS contributed to drafting the text and preparing the figures.

## Conflict of Interest

Dr. Bronte‐Stewart serves on a clinical advisory board for Medtronic PLC which supplied investigative sensing neurostimulators.

## References

[1] Goetz CG , Stebbins GT , Blasucci LM . Differential progression of motor impairment in levodopa‐treated Parkinson’s disease. Mov Disord. 2000;15(3):479‐484. 10830412 10.1002/1531-8257(200005)15:3<479::AID-MDS1009>3.0.CO;2-P

[2] Simuni T , Siderowf A , Lasch S , et al. Longitudinal change of clinical and biological measures in early Parkinson’s disease: Parkinson’s progression markers initiative cohort. Mov Disord. 2018;33(5):771‐782. 29572948 10.1002/mds.27361 PMC6001458

[3] Chahine LM , Siderowf A , Barnes J , et al. Predicting progression in Parkinson’s disease using baseline and 1‐year change measures. J Parkinsons Dis. 2019;9(4):665‐679. 31450510 10.3233/JPD-181518 PMC6839498

[4] Bergman H , Wichmann T , Karmon B , DeLong MR . The primate subthalamic nucleus. II. Neuronal activity in the MPTP model of Parkinsonism. J Neurophysiol. 1994;72(2):507‐520. 7983515 10.1152/jn.1994.72.2.507

[5] Brown P . Oscillatory nature of human basal ganglia activity: relationship to the pathophysiology of Parkinson’s disease. Mov Disord. 2003;18(4):357‐363. 12671940 10.1002/mds.10358

[6] Shreve LA , Velisar A , Malekmohammadi M , et al. Subthalamic oscillations and phase amplitude coupling are greater in the more affected hemisphere in Parkinson’s disease. Clin Neurophysiol. 2017;128(1):128‐137. 27889627 10.1016/j.clinph.2016.10.095

[7] Kehnemouyi YM , Wilkins KB , Anidi CM , Anderson RW , Afzal MF , Bronte‐Stewart HM . Modulation of beta bursts in subthalamic sensorimotor circuits predicts improvement in bradykinesia. Brain. 2021;144(2):473‐486. 33301569 10.1093/brain/awaa394 PMC8240742

[8] Neumann W‐J , Degen K , Schneider G‐H , et al. Subthalamic synchronized oscillatory activity correlates with motor impairment in patients with Parkinson’s disease. Mov Disord. 2016;31(11):1748‐1751. 27548068 10.1002/mds.26759 PMC5120686

[9] Steiner LA , Neumann W‐J , Staub‐Bartelt F , et al. Subthalamic beta dynamics mirror Parkinsonian bradykinesia months after neurostimulator implantation. Mov Disord. 2017;32(8):1183‐1190. 28639263 10.1002/mds.27068 PMC5575541

[10] Feingold J , Gibson DJ , DePasquale B , Graybiel AM . Bursts of beta oscillation differentiate postperformance activity in the striatum and motor cortex of monkeys performing movement tasks. Proc Natl Acad Sci USA. 2015;112(44):13687‐13692. 26460033 10.1073/pnas.1517629112 PMC4640760

[11] Tinkhauser G , Pogosyan A , Little S , et al. The modulatory effect of adaptive deep brain stimulation on beta bursts in Parkinson’s disease. Brain. 2017;140(4):1053‐1067. 28334851 10.1093/brain/awx010 PMC5382944

[12] Anidi C , O’Day JJ , Anderson RW , et al. Neuromodulation targets pathological not physiological beta bursts during gait in Parkinson’s disease. Neurobiol Dis. 2018;120:107‐117. 30196050 10.1016/j.nbd.2018.09.004 PMC6422345

[13] Deffains M , Iskhakova L , Katabi S , Israel Z , Bergman H . Longer β oscillatory episodes reliably identify pathological subthalamic activity in Parkinsonism. Mov Disord. 2018;33(10):1609‐1618. 30145811 10.1002/mds.27418

[14] Anderson RW , Kehnemouyi YM , Neuville RS , et al. A novel method for calculating beta band burst durations in Parkinson’s disease using a physiological baseline. J Neurosci Methods. 2020;343:108811. 32565222 10.1016/j.jneumeth.2020.108811 PMC7437353

[15] Dorval AD , Muralidharan A , Jensen AL , Baker KB , Vitek JL . Information in pallidal neurons increases with Parkinsonian severity. Parkinsonism Relat Disord. 2015;21(11):1355‐1361. 26433544 10.1016/j.parkreldis.2015.09.045 PMC4631644

[16] Muralidharan A , Jensen AL , Connolly A , et al. Physiological changes in the pallidum in a progressive model of Parkinson’s disease: are oscillations enough? Exp Neurol. 2016;279:187‐196. 26946223 10.1016/j.expneurol.2016.03.002 PMC4920549

[17] Wang J , Johnson LA , Jensen AL , et al. Network‐wide oscillations in the parkinsonian state: alterations in neuronal activities occur in the premotor cortex in Parkinsonian nonhuman primates. J Neurophysiol. 2017;117(6):2242‐2249. 28228579 10.1152/jn.00011.2017 PMC5461662

[18] Haumesser JK , Beck MH , Pellegrini F , et al. Subthalamic beta oscillations correlate with dopaminergic degeneration in experimental Parkinsonism. Exp Neurol. 2021;335:113513. 33148526 10.1016/j.expneurol.2020.113513

[19] Mallet N , Pogosyan A , Sharott A , et al. Disrupted dopamine transmission and the emergence of exaggerated beta oscillations in subthalamic nucleus and cerebral cortex. J Neurosci. 2008;28(18):4795‐4806. 18448656 10.1523/JNEUROSCI.0123-08.2008 PMC6670450

[20] Trager MH , Koop MM , Velisar A , et al. Subthalamic beta oscillations are attenuated after withdrawal of chronic high frequency neurostimulation in Parkinson’s disease. Neurobiol Dis. 2016;96:22‐30. 27553876 10.1016/j.nbd.2016.08.003

[21] Eusebio A , Thevathasan W , Doyle Gaynor L , et al. Deep brain stimulation can suppress pathological synchronisation in Parkinsonian patients. J Neurol Neurosurg Psychiatry. 2011;82(5):569‐573. 20935326 10.1136/jnnp.2010.217489 PMC3072048

[22] Whitmer D , de Solages C , Hill B , Yu H , Henderson JM , Bronte‐Stewart H . High frequency deep brain stimulation attenuates subthalamic and cortical rhythms in Parkinson’s disease. Front Hum Neurosci. 2012;6:155. 22675296 10.3389/fnhum.2012.00155 PMC3366347

[23] Bronte‐Stewart H , Barberini C , Koop MM , Hill BC , Henderson JM , Wingeier B . The STN beta‐band profile in Parkinson’s disease is stationary and shows prolonged attenuation after deep brain stimulation. Exp Neurol. 2009;215(1):20‐28. 18929561 10.1016/j.expneurol.2008.09.008

[24] Kühn AA , Tsui A , Aziz T , et al. Pathological synchronisation in the subthalamic nucleus of patients with Parkinson’s disease relates to both bradykinesia and rigidity. Exp Neurol. 2009;215(2):380‐387. 19070616 10.1016/j.expneurol.2008.11.008

[25] Wingeier B , Tcheng T , Koop MM , Hill BC , Heit G , Bronte‐Stewart HM . Intra‐operative STN DBS attenuates the prominent beta rhythm in the STN in Parkinson’s disease. Exp Neurol. 2006;197(1):244‐251. 16289053 10.1016/j.expneurol.2005.09.016

[26] Dembek TA , Roediger J , Horn A , et al. Probabilistic sweet spots predict motor outcome for deep brain stimulation in Parkinson disease. Ann Neurol. 2019;86(4):527‐538. 31376171 10.1002/ana.25567

[27] Neumann W‐J , Staub‐Bartelt F , Horn A , et al. Long term correlation of subthalamic beta band activity with motor impairment in patients with Parkinson’s disease. Clin Neurophysiol. 2017;128(11):2286‐2291. 29031219 10.1016/j.clinph.2017.08.028 PMC5779610

[28] Chen Y , Gong C , Tian YE , et al. Neuromodulation effects of deep brain stimulation on beta rhythm: a longitudinal local field potential study. Brain Stimul. 2020;13(6):1784‐1792. 33038597 10.1016/j.brs.2020.09.027

[29] Hacker ML , DeLong MR , Turchan M , et al. Effects of deep brain stimulation on rest tremor progression in early stage Parkinson disease. Neurology. 2018;91(5):e463‐e471. 29959266 10.1212/WNL.0000000000005903 PMC6093763

[30] Liang GS , Chou KL , Baltuch GH , et al. Long‐term outcomes of bilateral subthalamic nucleus stimulation in patients with advanced Parkinson’s disease. Stereotact Funct Neurosurg. 2006;84(5‐6):221‐227. 17063043 10.1159/000096495

[31] Østergaard K , Aa Sunde N . Evolution of Parkinson’s disease during 4 years of bilateral deep brain stimulation of the subthalamic nucleus. Mov Disord. 2006;21(5):624‐631. 16283616 10.1002/mds.20776

[32] Schüpbach WMM , Chastan N , Welter ML , et al. Stimulation of the subthalamic nucleus in Parkinson’s disease: a 5 year follow up. J Neurol Neurosurg Psychiatry. 2005;76(12):1640‐1644. 16291886 10.1136/jnnp.2005.063206 PMC1739461

[33] Hacker ML , Turchan M , Heusinkveld LE , et al. Deep brain stimulation in early‐stage Parkinson disease: five‐year outcomes. Neurology. 2020;95(4):e393‐e401. 32601120 10.1212/WNL.0000000000009946 PMC7455319

[34] Zibetti M , Merola A , Rizzi L , et al. Beyond nine years of continuous subthalamic nucleus deep brain stimulation in Parkinson’s disease. Mov Disord. 2011;26(13):2327‐2334. 22012750 10.1002/mds.23903

[35] Fasano A , Romito LM , Daniele A , et al. Motor and cognitive outcome in patients with Parkinson’s disease 8 years after subthalamic implants. Brain. 2010;133(9):2664‐2676. 20802207 10.1093/brain/awq221

[36] Merola A , Zibetti M , Angrisano S , et al. Parkinson’s disease progression at 30 years: a study of subthalamic deep brain‐stimulated patients. Brain. 2011;134(Pt 7):2074‐2084. 21666262 10.1093/brain/awr121

[37] Castrioto A , Lozano AM , Poon YY , Lang AE , Fallis M , Moro E . Ten‐year outcome of subthalamic stimulation in Parkinson disease: a blinded evaluation. Arch Neurol. 2011;68(12):1550‐1556. 21825213 10.1001/archneurol.2011.182

[38] Brontë‐Stewart H , Louie S , Batya S , Henderson JM . Clinical motor outcome of bilateral subthalamic nucleus deep‐brain stimulation for Parkinson’s disease using image‐guided frameless stereotaxy. Neurosurgery. 2010;67(4):1088‐1093. doi: 10.1227/neu.0b013e3181ecc887 20881573

[39] Syrkin‐Nikolau J , Koop MM , Prieto T , et al. Subthalamic neural entropy is a feature of freezing of gait in freely moving people with Parkinson’s disease. Neurobiol Dis. 2017;108:288‐297. 28890315 10.1016/j.nbd.2017.09.002 PMC6386531

[40] Horn A , Li N , Dembek TA , et al. Lead‐DBS v2: towards a comprehensive pipeline for deep brain stimulation imaging. NeuroImage. 2019;184:293‐316. 30179717 10.1016/j.neuroimage.2018.08.068 PMC6286150

[41] Avants BB , Tustison NJ , Song G , Cook PA , Klein A , Gee JC . A reproducible evaluation of ANTs similarity metric performance in brain image registration. NeuroImage. 2011;54(3):2033‐2044. 20851191 10.1016/j.neuroimage.2010.09.025 PMC3065962

[42] Husch A , Petersen MV , Gemmar P , Goncalves J , Hertel F . PaCER ‐ a fully automated method for electrode trajectory and contact reconstruction in deep brain stimulation. Neuroimage Clin. 2018;17:80‐89. 29062684 10.1016/j.nicl.2017.10.004 PMC5645007

[43] Ewert S , Plettig P , Li N , et al. Toward defining deep brain stimulation targets in MNI space: a subcortical atlas based on multimodal MRI, histology and structural connectivity. NeuroImage. 2018;170:271‐282. 28536045 10.1016/j.neuroimage.2017.05.015

[44] Tinkhauser G , Pogosyan A , Tan H , Herz DM , Kühn AA , Brown P . Beta burst dynamics in Parkinson’s disease OFF and ON dopaminergic medication. Brain. 2017;140(11):2968‐2981. 29053865 10.1093/brain/awx252 PMC5667742

[45] Horn A , Neumann W‐J , Degen K , Schneider G‐H , Kühn AA . Toward an electrophysiological “sweet spot” for deep brain stimulation in the subthalamic nucleus. Hum Brain Mapp. 2017;38(7):3377‐3390. 28390148 10.1002/hbm.23594 PMC6867148

[46] Klimesch W . α‐band oscillations, attention, and controlled access to stored information. Trends Cogn Sci. 2012;16(12):606‐617. 23141428 10.1016/j.tics.2012.10.007 PMC3507158

[47] Cilia R , Cereda E , Akpalu A , et al. Natural history of motor symptoms in Parkinson’s disease and the long‐duration response to levodopa. Brain. 2020;143(8):2490‐2501. 32844196 10.1093/brain/awaa181 PMC7566883

[48] Nutt JG , Woodward WR , Carter JH , Gancher ST . Effect of long‐term therapy on the pharmacodynamics of levodopa. Relation to on‐off Phenomenon. Arch Neurol. 1992;49(11):1123‐1130. 1444877 10.1001/archneur.1992.00530350037016

[49] Temperli P , Ghika J , Villemure J‐G , Burkhard PR , Bogousslavsky J , Vingerhoets FJG . How do Parkinsonian signs return after discontinuation of subthalamic DBS? Neurology. 2003;60(1):78‐81. 12525722 10.1212/wnl.60.1.78

[50] Hauser RA , Holford NHG . Quantitative description of loss of clinical benefit following withdrawal of levodopa‐carbidopa and bromocriptine in early Parkinson’s disease. Mov Disord. 2002;17(5):961‐968. 12360545 10.1002/mds.10226

